# HLA-E Polymorphism Determines Susceptibility to BK Virus Nephropathy after Living-Donor Kidney Transplant

**DOI:** 10.3390/cells8080847

**Published:** 2019-08-07

**Authors:** Hana Rohn, Rafael Tomoya Michita, Sabine Schramm, Sebastian Dolff, Anja Gäckler, Johannes Korth, Falko M. Heinemann, Benjamin Wilde, Mirko Trilling, Peter A. Horn, Andreas Kribben, Oliver Witzke, Vera Rebmann

**Affiliations:** 1 Department of Infectious Diseases, West German Centre for Infectious Diseases (WZI), University Hospital Essen, University Duisburg-Essen, 45147 Essen, Germany; 2 Institute for Transfusion Medicine, University Hospital Essen, University Duisburg-Essen, 45147 Essen, Germany; 3 Post-Graduation Program in Genetics and Molecular Biology, Genetics Department, Universidade Federal do Rio Grande do Sul (UFRGS), Porto Alegre 91501-970, Brazil; 4 Department of Nephrology, University Hospital Essen, University Duisburg-Essen, 45147 Essen, Germany; 5 Institute for Virology, University Hospital Essen, University Duisburg-Essen, 45147 Essen, Germany

**Keywords:** BK virus, polyomavirus, nephropathy, human leukocyte antigen-E, kidney transplantation

## Abstract

Human leukocyte antigen (HLA)-E is important for the regulation of anti-viral immunity. BK polyomavirus (BKPyV) reactivation after kidney transplant is a serious complication that can result in BKPyV-associated nephropathy (PyVAN) and subsequent allograft loss. To elucidate whether HLA-E polymorphisms influence BKPyV replication and nephropathy, we determined the HLA-E genotype of 278 living donor and recipient pairs. A total of 44 recipients suffered from BKPyV replication, and 11 of these developed PyVAN. Homozygosity of the recipients for the HLA-E*01:01 genotype was associated with the protection against PyVAN after transplant (*p* = 0.025, OR 0.09, CI [95%] 0.83–4.89). Considering the time course of the occurrence of nephropathy, recipients with PyVAN were more likely to carry the HLA-E*01:03 allelic variant than those without PyVAN (Kaplan–Meier analysis *p* = 0.03; OR = 4.25; CI (95%) 1.11–16.23). Our findings suggest that a predisposition based on a defined HLA-E genotype is associated with an increased susceptibility to develop PyVAN. Thus, assessing HLA-E polymorphisms may enable physicians to identify patients being at an increased risk of this viral complication.

## 1. Introduction

Human leukocyte antigen (HLA)-E belongs to the non-classical major histocompatibility complex (MHC) class Ib molecules located on chromosome 6p21.3. In terms of function, HLA-E is special within the immune system because it acts as a key player exhibiting regulatory functions both in innate and adaptive immune responses [1].

HLA-E predominantly acts as an indicator for “missing-self” by continuously presenting peptides derived from signal sequences from HLA class Ia molecules. Infected or transformed cells, which have silenced HLA-I expression to evade from canonical CD8 T-cell recognition, do not produce such signal peptides. In absence of the signal peptides, HLA-E is not stabilized and does not reach the cell surface. NK cells sense the absence of HLA-E and become activated. Recently it became apparent that HLA-E can also bind and present antigenic peptides derived from pathogens such as HIV, hepatitis B and C viruses as well as cytomegalovirus [2], a fact implying that HLA-E plays an important role in anti-viral immunity mediated by T cells [3,4]. Being the cognate ligand for the C-type lectin CD94/NKG2 receptor family, HLA-E enables natural killer (NK) cells and cytotoxic T lymphocytes to monitor cell integrity because it is recognized by either the inhibitory CD94/NKG2A or the activating CD94/NKG2C receptor [5,6,7]. Moreover, HLA-E can be recognized by the stimulatory α/β T-cell receptor (TCR) expressed on the CD8+ T cells, resulting in cytotoxic elimination of target cells presenting foreign peptides presented by HLA-E [8].

In contrast to the highly polymorphic HLA-A, HLA-B, or HLA-C molecules, HLA-E exhibits fewer allelic variants [9]. To date, only 27 HLA-E alleles have been reported, most of which are found in neglectable frequencies in the population or do not encode functional proteins at all [10]. The two most prevalent allotypes in the Caucasian population are HLA-E*01:01 (HLA-E107R) and HLA-E*01:03 (HLA-E107G), which are found with nearly equal frequencies [11]. Although the two corresponding proteins only differ by an arginine (HLA-E*01:01) or a glycine (HLA-E*01:03) at position 107 [12], functional differences have been reported. HLA-E*01:03 has been reported to exhibit a higher affinity for available peptides, and for this reason its cell surface expression is significantly increased compared to HLA-E*01:01 [11,13]. Studies of viral diseases have shown that the various HLA-E genotypes affect a clinical outcome [14,15,16].

Little is known about the relevance of HLA-E concerning viral infection and reactivation after solid organ transplant. Viruses are the leading cause of infections and mortality after transplant. In particular, the reactivation of otherwise mostly subclinical, opportunistic viruses from pre-existing latency reservoirs is clinically relevant during immunosuppression. BK polyomavirus (BKPyV), a small non-enveloped double-stranded DNA virus, has emerged as one of the most challenging pathogens after kidney transplant, causing severe allograft dysfunction and graft loss. Approximately 80% to 95% of the human population are persistently infected with BKPyV; the infection mostly occurs in healthy adults [17,18,19]. However, the immunosuppression necessary after kidney transplant enables the virus to reactivate. Viral replication can occur in as many as 60% of recipients [20], and can progress to the most severe form of BKPyV invasive kidney disease, polyomavirus-associated nephropathy (PyVAN).

PyVAN is linked to kidney malfunction and rejection, with a significant risk of allograft loss in as many as 60% of the cases [19,21,22,23]. Unfortunately, no direct antiviral treatment is approved for BKPyV replication, and the current recommendation for BKPyV management and PyVAN treatment is restricted to a reduction of immunosuppression, leading to a substantial risk of acute rejection. Little is known about the pathogenesis of BKPyV, and it is not clear which factors determine the clinical course of PyVAN. From a clinical perspective, the identification of genetic biomarkers that influence the course of BKPyV infection may allow early risk stratification to prevent the progression of PyVAN.

Thus, considering the functional differences between the HLA-E allelic variants, we hypothesized that these allelic variants may affect the clinical occurrence and onset of PyVAN after kidney transplant and may serve as a prognostic parameter for determining which patients are at risk.

## 2. Materials and Methods

### 2.1. Study Population, BKPyV and PyVAN Screening

A total of 278 living-donor kidney transplant recipients and their 278 corresponding donors from the living-donor kidney program at the University Hospital Essen, Germany, were enrolled in this retrospective study. Transplant procedures were performed between 2005 and 2017. Informed consent was obtained from all patients in accordance with the Declaration of Helsinki, and the local ethics committee approved the study (approval number 12-5312-BO). All patients underwent regular follow-up examinations. An exclusion criterion was treatment with an mTOR inhibitor, since clinical data indicate that the use of mTOR-based immunosuppressive regimens may be protective against BKPyV replication [22,24,25,26]. The following data were collected: demographic and transplant-related characteristics of recipients and donors (Table 1), the occurrence of BKPyV, and the occurrence of biopsy-proven PyVAN.

Screening for BKPyV replication after transplant evolved during the course of the study in accordance with the introduction of new clinical guidelines. Initially, only patients with graft dysfunction were tested for BKPyV replication. Starting from 2010, the screening for BKPyV replication after kidney transplant was performed according to the 2009 clinical practice guideline Kidney Disease: Improving Global Outcomes (KDIGO). In addition to scheduled BKPyV screening, patients´ serum was tested for BKPyV replication once allograft dysfunction was detected. All samples were analyzed by quantitative real-time polymerase chain reaction (RT-PCR) with a lower limit of quantification of 400 copies per milliliter. BKPyV reactivation was defined as viremia when RT-PCR detected BKPyV DNA in patients´ serum.

Occurrence of PyVAN was assessed during the first three years after transplantation. PyVAN was diagnosed, if found in at least one kidney biopsy specimen judged positive by an experienced renal pathologist according to standard criteria [27]. Kidney biopsies were performed only upon clinical indication of PyVAN, like evidence of graft dysfunction or high levels of BKPyV viremia. Besides BKPyV, cytomegalovirus (CMV) represents an important viral pathogen that negatively affects allograft survival [28,29,30,31]. We have previously reported that the polymorphism of HLA-E is associated with CMV infection after kidney transplantation [16]. To evaluate possible interactions between these two viral infections with respect to the HLA-E polymorphism, we determined the rate of CMV infections for the present cohort as defined by international recommendations [32]. Standard induction immunosuppression consisted of treatment with a basiliximab-based regimen (which blocks the IL-2 receptor CD25) and a calcineurin inhibitor (CNI) in combination with steroids and an antiproliferative drug (mycophenolic acid or azathioprine).

### 2.2. HLA-E Genotyping

Recipients and corresponding donors were typed for HLA-E with a sequence-specific primer-polymerase chain reaction (SSP-PCR) method as described previously [33,34]. Genomic DNA was isolated from buffy-coats of peripheral blood using QIAamp^®^ DNA Blood Mini Kit (QIAGEN GmbH, D-40724 Hilden, Germany). The SSP-PCR method was performed with the following conditions: 50 ng of genomic DNA was amplified in a final reaction volume of 10 µL containing 3 µL PCR Master Mix (Olerup^®^ SSP AB, Stockholm, Sweden), 0.6 units of Taq DNA Polymerase (QIAGEN GmBH), 15 pmol of detection primers and 15 pmol of each positive control primer. The initial denaturation of the sequence-specific products was performed for 2 min at 94 °C, followed by a two stage PCR program: 10 cycles of 10 s at 94 °C and 20 s at 65 °C, as well as 20 relaxed cycles of 10 s at 94 °C, of 1 min at 61 °C and of 30 s at 72 °C. HLA-E alleles were identified at the resolution level of the second field. Human growth hormone was used as a positive control for PCR amplification. HLA-E genotype distributions in the two groups matched expectations according to the Hardy–Weinberg equilibrium.

### 2.3. Prediction of HLA-E Affinity for BKPyV Derived Peptides

The non-classical HLA-E molecule exhibits preferential binding to a highly conserved set of nonameric signal peptides derived from leader sequences of other HLA-class I molecules. However, HLA-E is also able to present pathogen-derived antigens [2], hence antigens derived from CMV, human immunodeficiency virus (HIV) and other pathogens give rise to peptides, which are loaded onto HLA-E molecules [14,35]. To our knowledge, HLA-E restricted peptides encoded by BKPyV have not been described to date. To identify peptide sequences derived from BKPyV antigens that may bind to HLA-E with high affinity and thus potentially representing immunogenic T-cell epitopes, we applied the machine-learning bioinformatics algorithm NetMHC 4.0 (http.//www.cbs.dtu.dk/services/NetMHC/index.php) [36,37]. NetMHC 4.0 is a method trained on both binding affinity and eluted ligand data. The algorithm uses an allele-specific approach, whereby separate predictors are trained for each MHC allele; the input to the model is the peptide of interest (in our case derived from BKPyV) [36]. The following BKPyV-derived antigens were assessed for HLA-E binding potential: large-T and small-T antigen, the major capsid proteins VP1 and VP2, and the agnoprotein. In order to compare the affinities of putative BKPyV-derived peptides predicted by the NetMHC 4.0 algorithm, functionally well-defined HLA-E binding peptides derived from the classical HLA class I molecule HLA-G and HLA-Cw*3, the heath shock protein 60, CMV proteins (pUL40, pUL18, pUL83, and pUL123), and two other pathogen-derived peptides (viz., the HIV gag protein and mycobacterium tuberculosis enoyl-[acyl-carrier-protein] reductase) were also determined [8,38,39,40,41]. We only took “strong binding peptides” as defined by the NetMHC 4.0 algorithm into consideration. Strong binding peptides are defined by an affinity threshold of 50,000 nM as well as a percentile rank assignment within <0.5 being a predicted affinity compared to a set of 400,000 random natural peptides. Solely the HLA-E*01:01 allele is present in NetMHC 4.0; thus we were able to determine the peptide affinity for this allelic variant only.

### 2.4. Statistical Analysis

Statistical analyses were performed using the SPSS 21.0 software (IBM corp. Released 2012, IBM SPSS Statistic for Windows, Armonk, NY, USA). Baseline characteristics of donors and recipients were compared with two-sided Fisher’s exact test or the Wilcoxon rank-sum test, as appropriate. The contribution of allelic variants as risk factors for PyVAN was evaluated by Fisher’s exact test. Joint genotype analysis was performed by Mantel–Haenszel test. The occurrence of PyVAN was estimated by the Kaplan–Meier method, and estimates were compared with the log-rank test. A two-sided *p*-value of 0.05 or lower was considered statistically significant.

## 3. Results

### 3.1. HLA-E*01:01 May Be Able to Present Peptide Sequences Derived from BKPyV

We were able to identify BKPyV-derived peptides that may be presented by the HLA-E*01:01 allelic variant using the machine-learning bioinformatics algorithm NetMHC 4.0 (National Institute of Allergy and Infectious Diseases, National Institutes of Health, Bethesda, MD, USA). Table 2, Table 3, Table 4 and Table 5 contains the identified high and moderate affinity peptide sequences derived from BKPyV antigens (large-T and small-t antigen, VP1, VP2 and agnoprotein) which may be presented to immune effector cells by HLA-E*01:01.

The affinities of putative nonameric BKPyV-derived peptides predicted by the NetMHC 4.0 algorithm were compared to the HLA-E-binding peptide repertoire reported in the literature [8,38,39,40,41] (Supplementary Table S1a–i). Table 6 summarizes the results of the binding affinities of the identified peptide motifs with the highest affinity as predicted by the NetMHC 4.0 algorithm.

Leader peptide sequences of HLA-Cw*3, the CMV homologue pUL40 [35], and HLA-G molecules had the highest predicted binding affinity to HLA-E. The predicted binding affinities of the BKPyV-derived antigens ranged between these and other well-characterized peptide sequences. It is noteworthy that the primary anchor residues among the leader peptides sequences are largely conserved at the canonical position 2 Met (methionine) and position 9 Leu (leucine) [6,9]. Three out of four identified BKPyV HLA-E binding motifs have Leu at position 9 and the BK polyomavirus major capsid protein VP2 has also Met at position 2.

### 3.2. HLA-E*01:01 Homozygosity Exerts Protection against PyVAN

HLA-E allelic frequencies in our cohort were similar among recipients (HLA-E*01:01 294 of 556 [52.9%]; HLA-E*01:03 262 of 556 [47.1%]) and donors (HLA-E*01:01 282 of 556 [50.7%]; HLA-E*01:03 274 of 556 [49.3%]). No other HLA-E variants were detected. The observed allelic distribution of HLA-E was in accordance with expectations indicated by the Hardy–Weinberg equilibrium.

In total, 44 recipients (15.8%) tested positive for BKPyV viremia; 23 of these exhibited high levels of BKPyV viremia (>10^4^ copies per milliliter). Eleven BKPyV-positive recipients had at least one biopsy-proven PyVAN during the first three years after kidney transplant. Three out of the eleven patients experienced CMV replication prior to the development of a BK virus-associated nephropathy. All three patients, in which the CMV replication preceded the BK virus-associated nephropathy, were HLA-E*01:03 carriers.

With respect to HLA-E genotypes, there was no association of HLA-E genotypes of the donor and the occurrence of PyVAN (Table 7).

However, the recipient HLA-E*01:01 homozygous state was associated with protection against PyVAN, (*p* = 0.025, odds ratio [OR] 0.09, 95% confidence interval [CI] 0.83–4.89; Table 7). By combining BK virus nephropathy with the occurrence of CMV replication, the same observation was made (Table 8).

**Figure 1:**
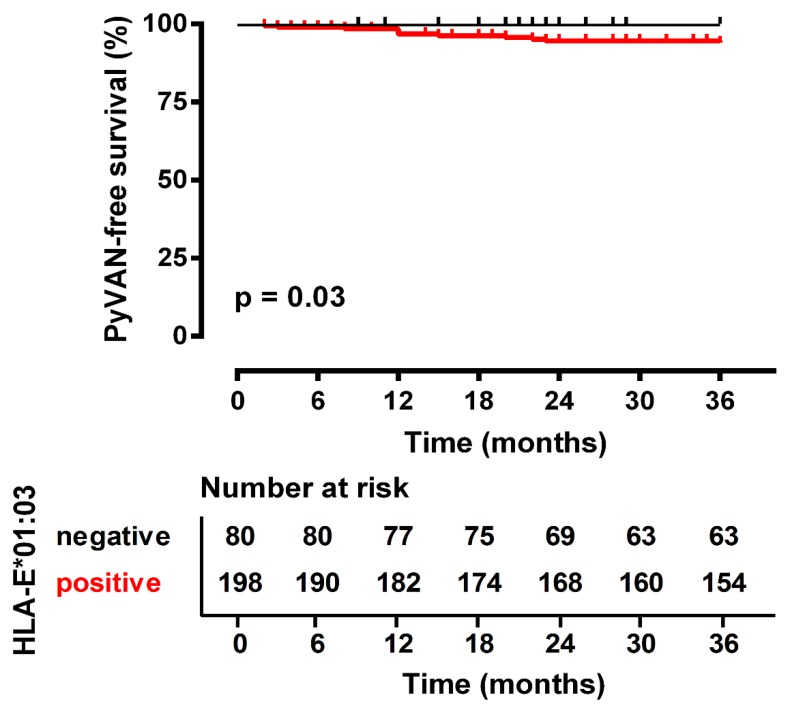
Association between the PyVAN-free survival and the HLA-E*01:03 carrier status of the transplant recipient during the first three years after living-donor kidney transplant. Recipients carrying the HLA-E*01:03 allelic variant were significantly more susceptible to develop PyVAN.

Considering the time course of PyVAN occurrence, the results of Kaplan–Meier analysis combined with those of the log-rank test (Figure 1) indicate that recipients carrying the HLA-E*01:03 allele were at significantly higher risk of PyVAN during the first three years after kidney transplant as compared to non-carriers (*p* = 0.03; OR = 4.25; 95% CI 1.11–16.23). Of note, all 11 recipients with PyVAN carried at least one HLA-E*01:03 allele.

With regard to HLA-E polymorphism of donor or recipient, we found no association with the occurrence or level of BKPyV viremia.

## 4. Discussion

To gain insights into BKPyV replication after kidney transplant, we evaluated the role of HLA-E genotypes in a large cohort of living kidney transplant pairs. To our knowledge, this is the first study demonstrating that the HLA-E polymorphism is associated with the pathogenesis of PyVAN after kidney transplant. Our study indicates that considering the time course of PyVAN occurrence, the prevalence of the HLA-E*01:03 allelic variant among living-donor kidney transplant recipients with PyVAN was significantly higher than that among recipients with no PyVAN. In addition, the homozygous status of the HLA-E*01:01 allele in the recipient is associated with protection against PyVAN.

This finding is in line with those of the previous studies attributing the distinctive susceptibility of patients with the HLA-E polymorphism to various viral infections. Recently, we found that HLA-E*01:03 carrier status is associated with cytomegalovirus infection after kidney transplant. Similarly, Schulte et al. reported that the homozygous HLA-E*01:01 genotype exerts a protective effect against Hepatitis C virus [16,43].

Despite the highly negative impact of BKPyV on patient and allograft survival, little is known about the immunology of BKPyV infection. Patients with a high BKPyV viral load are particularly likely to develop PyVAN and to experience allograft loss. The implementation of regular BKPyV monitoring has become a useful tool allowing the detection of patients at risk of PyVAN. The current treatment strategy against sustained BKPyV replication is a stepwise reduction or modification of the immunosuppression so that the endogenous immune control of the virus is gradually restored, thereby potentially preventing the development of PyVAN. However, this approach must be balanced against the increasing risk of allograft rejection and is not always successful. The identification of additional predictive markers indicating which patients are especially prone to PyVAN is therefore important. In combination with viral molecular monitoring, the analysis of specific immune biomarkers can make a decisive contribution in supporting the surveillance and treatment of kidney transplant recipients at risk of PyVAN. This approach will allow new preventive measures, alternative treatment strategies, and patient-tailored immunosuppression. As is the case for cytomegalovirus infection, clinical evidence indicate that the use of mTOR-based immunosuppressive regimens may be protective against BKPyV replication [24,26].

Only three patients had a CMV and BKPyV co-replication in this cohort. In such cases of combined BK virus nephropathy and CMV replication, it became evident that both events were associated with HLA-E*01:03 recipient carrier status. However, we cannot exclude that CMV additionally promotes development of BK virus nephropathy. After transplantation, CMV DNAemia has been associated with a general state of over-immunosuppression, potentially increasing risk of complications elicited by opportunistic infections [44]. Interestingly, however, Reischig et al. recently reported that the risk of BK viremia as well as PyVAN is decreased in patients who experienced CMV DNAemia [45]. At this stage, it is impossible to clarify if CMV affected the occurrence and/or severity of PyVAN in the three cases.

The host immune response is essential in controlling the viral carrier state. Increasing amount of evidences suggest that the immunomodulatory molecule HLA-E plays an important role in viral immunity. HLA-E is broadly expressed at low levels, and its expression can be up-regulated during cellular stress, such as viral infection. HLA-E is special within the immune system because it not only exhibits regulatory functions in innate and adaptive immune responses, but also promotes activating as well as inhibitory signals. These differences in HLA-E functions lead to differential effects on T- and NK-cell activity and depend on three determinants: (i) the nature of the presented peptides, (ii) the cognate receptor repertoire, and (iii) the expression level of the HLA-E alleles. HLA-E preferentially binds leader peptides sequences derived from classical HLA-class I molecules. In addition to presenting self-antigens, HLA-E can bind several nonameric peptides of viral origin [35,46,47]. It has been shown that the binding repertoire of HLA-E is broad and that the diversity of HLA-E binding motifs can considerably differ from the leader peptides sequences of classical HLA-class I molecules [35,41]. To date no study has shown that HLA-E binds BKPyV-specific peptides, however, the bioinformatics algorithm NetMHC 4.0 [36,37] has identified several peptide sequences derived from BKPyV early antigens (large-T and small-t) and late antigens (VP1 and VP2) that may bind to HLA-E with high affinity. The predicted binding affinity of the BKPyV-derived antigens in our study falls between the values of the leader peptide sequences of HLA-G and HLA-Cw*3 molecules and the HIV Gag-derived peptide. Of note the HIV Gag-peptide is a NetMHC predicted epitope being a homologous to the Simian immunodeficiency virus (SIV) Gag-derived peptide, which has recently been shown to comprise one of the two supertopes recognized by 100% of rhesus macaques vaccinated against SIV [48].

The presented peptide is of great importance because the interaction between the HLA-E-peptide and the cognate activating CD94/NKG2C or inhibitory CD94/NKG2A receptors expressed on the surface of NK cells and certain T cells has been described as peptide-sensitive [49,50]. Analysis of amino acid preferences for HLA-E binding at each anchor position demonstrated a clear preference for methionine (Met) at positions 2 and a leucine (Leu) at position 9 [9,35,42]. Three of the four BKPyV-derived peptide sequences identified in this study have a Leu at position 9, and one also has a Met at position 2, rendering HLA-E binding rather likely. Intriguingly, the activating receptor CD94/NKG2C is found less frequently and binds to the ligand HLA-E with lower affinity and stricter peptide selectivity than the inhibitory CD94/NKG2A receptor [51,52]. HLA-presented peptides are also of importance for the regulation of adaptive NK-cells expressing the activating CD94/NKG2C receptor [53,54,55,56,57,58]. Recently, Horowitz A et al. demonstrated that the binding affinity of the presented HLA-E peptide by affection of HLA-E cell surface expression impacts on the NK cell education through its influence on HLA-E cell surface disposition. As a result of enhanced HLA-E expression, NK cell education is dominated by CD94/NKG2A, whereas in individuals with peptide motifs with low binding affinity to HLA-E, NK cell education is dominated by inhibitory killer-cell immunoglobulin-like receptors (KIRs) [59]. Moreover, viral infections may imprint on the CD94/NKG2 receptor compartment, as has been shown for cytomegalovirus and Hepatitis C virus [60]. Studies using mice infected with polyomavirus (PyV) found that CD94/NKG2A expression is rapidly induced on antiviral CD8+ T-cells, thereby reducing the cytotoxic activity of PyV-specific CD8+ T-cells [61,62,63,64]. Since the HLA-E*01:03 variant consistently shows higher cell surface expression compared to HLA-E*01:01, the baseline engagement of the inhibitory CD94/NKG2A might even be higher during viral infection, thus promoting viral immune escape. With regard to HLA-E*01:01, herein described potential HLA-E*01:01-specific T cell epitopes might be recognized by CD8+ T cells leading to immune activation.

In our cohort, the recipient homozygous genotype HLA-E*01:01 was associated with protection against PyVAN, whereas HLA-E*01:03 carrier status in recipients was associated with an increased risk of PyVAN after kidney transplant. In the context of transplant, it is necessary to take recipient- and donor-derived genetic factors that may influence the outcome of viral infection into account. Although the two HLA-E alleles differ only by a single amino acid, this subtle difference has important functional implications, because HLA-E*01:03 proteins exhibit consistently higher surface expression than does HLA-E*01:01 [11,65]. This discrepancy in cell surface expression suggests differences in the effective engagement of the CD94/NKG2A or C receptors expressed on NK and T cells. With a dominant presence of the inhibitory CD94/NKG2A receptor on effector cells, the HLA-E*01:03 variant may be related to a more pronounced dampening of inhibitory receptor responses and, thus, to insufficient immune control of viruses like CMV and BKPyV. Conversely, the presence of the homozygous HLA-E*01:01 variant may result in a lower probability of interaction with its cognate inhibitory CD94/NKG2A receptor, a condition resulting in increased susceptibility to lysis mediated by effector cells.

However, this single-center analysis has its limitations due to the retrospective nature of the study and the low incidence of PyVAN in our cohort, which did not allow multivariate analysis. Additionally, our conclusions on the effects of HLA-E polymorphism on BKPyV viremia are limited due to the changes in the screening procedure for BKPyV viremia during study period. Nevertheless, our results provide a rational for future prospective clinical studies and detailed mechanistic analyses.

## 5. Conclusions

The data presented here suggest that the HLA-E genetic predisposition of the recipients may influence the susceptibility to PyVAN. Thus, testing for the HLA-E polymorphism may enable physicians to determine which patients are at risk of PyVAN.

## Figures and Tables

**Table 1:** Patient characteristics at baseline.

	A	B	C	
	Total	HLA-E*01:03 Carrier#	HLA-E*01:03 Non-Carrier	*p*-Value B vs. C
**Donor**	**N = 278**	**N = 211**	**N = 67**	
Sex (men/women)	115/163	89/122	26/41	0.67 ^a^
Age (y) ±SD	51.41 ± 10.03	51.99 ± 10.06	43.30 ± 9.83	0.10 ^b^
**Recipient**	**N = 278**	**N = 198**	**N = 80**	
Sex (men/women)	162/116	116/82	46/34	0.87 ^a^
Age (y) ±SD	41.06 ± 15.43	40.46 ± 15.42	42.53 ± 15.47	0.32 ^b^
**KTx Related Variables**				
HLA A, B mismatches, mean ± SD	1.97 ± 1.14	1.83 ± 1.1	2.31 ± 1.17	0.002 **^b^
HLA-DR mismatch, mean ± SD	1.12 ± 0.62	1.09 ± 0.60	1.19 ± 0.66	0.22 ^b^
**Panel of Antibodies (%)**				
0%	244	172	72	0.55 ^a^
1–10%	14	9	5	0.55 ^a^
10–50%	14	12	2	0.36 ^a^
>50%	6	5	1	0.68 ^a^
Cold ischemia time, median, in minutes (range)	134.99 ± 48.90	136.07 ± 48.63	131.32 ± 48.86	0.57 ^b^
Warm ischemia time, median, in minutes (range)	19.97 ± 6.87	19.38 ± 5.84	21.46 ± 8.83	0.057 ^b^
**Immunosuppressive Therapy**				
ATG-based induction therapy, yes/no	13/265	9/189	4/76	1 ^a^
CNI administration, yes/no	278/0	198/0	80/0	nd
MMF co-administration, yes/no	260/18	189/9	75/5	0.55 ^a^
Steroid co-administration, yes/no	278/0	198/0	80/0	nd
**PyVAN and CMV**				
PyVAN, yes/no	11/267	11/187	0/80	0.031 *^a^
CMV positive recipient	159/119	116/82	43/37	0.46 ^a^
CMV positive donor	177/101	128/70	49/31	0.59 ^a^
CMV Infection, yes/no	38/240	32/166	6/74	0.057 ^a^
PyVAN and CMV infection, yes/no	3/275	3/195	0/80	0.27 ^a^
PyVAN or CMV infection vs. no PyVAN and no CMV infection	46/232	40/158	6/74	0.01 ^a^

y: years; HLA: human leukocyte antigen; ATG: antithymocyte globulin; CNI: calcineurin inhibitor; MMF: mycophenolate mofetil; KTx: kidney transplant; PyVAN: BK polyomavirus associated nephropathy; CMV: cytomegalovirus; nd: not determined; SD: standard deviation. ^a^ Fisher’s exact test; ^b^ Mann–Whitney U test; # HLA E*01:03 carrier: HLA E*01:03/01:03 and HLA E*01:03/01:01genotype; HLA E*01:03 non-carrier: HLA E*01:01/01:01 genotype. * *p* < 0.05, ** *p* < 0.01.

**Table 2:** High and moderate affinity peptide sequences derived from BK polyomavirus (BKPyV) large T antigen and small t antigen that may be presented by the HLA-E*01:01 allelic variant as identified by the machine-learning bioinformatics algorithm NetMHC4.0. Position: residue number (starting from 0); peptide: amino acid sequence of the potential ligand.

Large-T and Small-t Antigen			
Position	Peptide	1 − log50k(aff)	Affinity (nM)
416	NVPKRRYWL	0.202	5619.93
568	RILQSGMTL	0.173	7666.40
454	PMERLTFEL	0.165	8432.78
557	SLQNSEFLL	0.160	8874.14
570	LQSGMTLLL	0.150	9850.58

**Table 3:** High and moderate affinity peptide sequences derived from BKPyV major capsid protein VP1 that may be presented by the HLA-E*01:01 allelic variant as identified by the machine-learning bioinformatics algorithm NetMHC 4.0. Position: residue number (starting from 0); peptide: amino acid sequence of the potential ligand.

BK Polyomavirus Major Capsid Protein VP1			
Position	Peptide	1 − log50k(aff)	Affinity (nM)
18	KEPVQVPKL	0.186	6716.19
237	TNTATTVLL	0.142	10,773.00
297	NPYPISFLL	0.139	11,067.54

**Table 4:** High affinity peptide sequences derived from BKPyV major capsid protein VP2 that may be presented by the HLA-E*01:01 allelic variant as identified by the machine-learning bioinformatics algorithm NetMHC 4.0. Position: residue number (starting from 0); peptide: amino acid sequence of the potential ligand.

BK Polyomavirus Major Capsid Protein VP2			
Position	Peptide	1 − log50k(aff)	Affinity(nM)
292	WMLPLLLGL	0.152	9685.81

**Table 5:** High and moderate affinity peptide sequences derived from BKPyV agnoprotein that may be presented by the HLA-E*01:01 allelic variations as identified by the machine-learning bioinformatics algorithm NetMHC 4.0. Position: residue number (starting from 0); peptide: amino acid sequence of the potential ligand.

BK polyomavirus agnoprotein			
Position	Peptide	1 − log50k(aff)	Affinity (nM)
46	SXXPESVMF	0.139	11,081.08
52	VMFCEPKNL	0.139	11,162.18

**Table 6:** The high affinity peptide motifs reported in the literature and derived from different self or foreign antigens as well as BKPyV-derived antigens that may be presented by HLA-E*01:01 allelic variations identified by the machine-learning bioinformatics algorithm NetMHC 4.0.

Derived from	Peptide	1 − log50k(aff)	Affinity (nM)
Human leukocyte antigen-Cw*3	VMAPRTLIL	0.589	85.69
CMV protein pUL40	VMAPRTLIL	0.589	85.69
Human leukocyte antigen-G	VMAPRTLFL	0.564	112.12
Mycobacterium tuberculosis enoyl-[acyl-carrier-protein] reductase [NADH]	RLPAKAPLL	0.547	134.77
CMV phosphorylated matrix protein pp65 (UL83)	VLPHETRLL	0.330	1405.23
CMV immediate-early protein 1 (pUL123)	VMLAKRPLI	0.303	1887.52
Human heat shock protein 60 (hsp60)	QMRPVSRVL	0.276	2530.17
CMV protein pUL18	SEPQCNPLL	0.273	2614.63
BK polyomavirus large-T and small-t antigen	NVPKRRYWL	0.202	5619.93
BK polyomavirus major capsid protein VP1	KEPVQVPKL	0.186	6716.19
Human immunodeficiency virus 1 Gag protein	RMYSPVSIL	0.173	7728.11
BK polyomavirus major capsid protein VP2	WMLPLLLGL	0.152	9685.81
BK polyomavirus agnoprotein	SXXPESVMF	0.139	11081.08

Methionine at amino acid positions 2 and leucine at position 9 amino acid have been described to be of functional relevance for HLA-E binding [9,35,42]. Peptide: amino acid sequence of the potential ligand.

**Table 7:** Genotype distribution of allele frequencies of HLA-E polymorphism in living-donor kidney transplant recipients (A) and corresponding donors (B) with respect to BK polyomavirus nephropathy (PyVAN).

**(A) Recipient** **HLA-E genotype**	**PyVAN** **N = 11**	**No PyVAN** **N = 267**	***p*-Value**	**OR**	**95% CI**
01:03/01:03	3 (27.3%)	61 (22.8%)	0.73	1.26	0.33–4.92
01:01/01:03	8 (72.7%)	126 (47.2%)	0.10	2.98	0.77–11.50
01:01/01:01	0 (0%)	80 (30%)	0.025 *	0.09	0.005–1.59
**Allele Frequencies**					
01:03	14	248	0.11	2.02	0.83–4.89
01:01	8	286			
**(B) Donor** **HLA-E genotype**	**PyVAN** **N = 11**	**No PyVAN** **N = 267**	***p*-Value**	**OR**	**CI (95%)**
01:03/01:03	1 (9.1%)	62 (23.2%)	0.27	0.33	0.04–2.63
01:01/01:03	9 (81.8%)	139 (52.1%)	0.052	4.14	0.89–19.55
01:01/01:01	1 (9.1%)	66 (24.7%)	0.24	0.3	0.04–2.42
**Allele Frequencies**					
01:03	11	263	0.77	1.1	0.47–2.7
01:01	10	271			

* *p* < 0.05.

**Table 8:** Genotype distribution of allele frequencies of HLA-E polymorphism in living-donor kidney transplant recipients (A) and corresponding donors (B) with respect to BK polyomavirus nephropathy (PyVAN) or cytomegalovirus (CMV) infection.

**(A) Recipient HLA-E genotype**	**PyVAN or CMV Infection** **N = 46**	**No PyVAN and no CMV Infection N = 267**	***p*-Value**	**OR**	**95% CI**
01:03/01:03	12 (26.1%)	52 (22.4%)	0.57	1.22	0.59–2.53
01:01/01:03	28 (60.9%)	106 (45.7%)	0.07	1.85	0.96–3.53
01:01/01:01	6 (13.0%)	74 (31.9%)	0.012 *	0.32	0.13–0.79
**Allele Frequencies**					
01:03	52	210	0.052	1.57	1.00–2.47
01:01	40	254			
**(B) Donor HLA-E genotype**	**PyVAN or CMV Infection** **N = 46**	**No PyVAN and no CMV Infection** **N = 267**	***p*-Value**	**OR**	**CI (95%)**
01:03/01:03	12 (9.1%)	51 (22.0%)	0.44	1.33	0.64–2.79
01:01/01:03	26 (81.8%)	122 (52.6%)	0.75	1.17	0.62–2.22
01:01/01:01	8 (9.1%)	59 (25.4%)	0.34	0.62	0.27–1.39
**Allele Frequencies**					
01:03	50	224	0.31	1.3	0.81–1.99
01:01	42	240			

* *p* < 0.05.

## Supplementary Materials

The following are available online at https://www.mdpi.com/2073-4409/8/8/847/s1, Table S1a-i: High- and moderate peptide sequences that may be presented by HLA-E*01:01 allelic variations (as identified by the machine-learning bioinformatics algorithm NetMHC 4.0) deriving from (a) CMV Protein UL-40, (b) CMV Protein UL-18, (c) CMV Phosphorylated matrix protein pp65 (UL83), (d) CMV immediate-early protein 1 (UL123), (e) Human leukocyte antigen-G, (f) Human leukocyte antigen-Cw*3, (g) Human heat shock protein (hsp60), (h) Human immundeficency virus 1 Gag protein, (i) Mycobacterium tuberculosis Enoyl -[acyl-carrier-protein]-reductase [NADH].
