# Supplementary material for: HLA-E Polymorphism Determines Susceptibility to BK Virus Nephropathy after Living-Donor Kidney Transplant
Source: Cells. 2019 Aug 7;8(8):847. doi: 10.3390/cells8080847 (PMC6721664; doi:10.3390/cells8080847)
Supplement: Supplementary file 1 [file cells-08-00847-s001.zip › cells-543425-supplementary.pdf]

Supplementary Tables 1a-1i

CMV Protein pUL40			
**Position**	**Peptide**	**1-log50k(aff)**	**Affinity (nM)**
14	VMAPRTLIL	0.589	85.69
171	KLRPATFGL	0.273	2610.87
96	HHPADNTLL	0.173	7724.60
28	CMRIRSLLC	0.133	11856.34

Supplementary Table 1a: High- and moderate-affinity peptide sequences derived from CMV Protein UL40 that may be presented by HLA-E*01:01 allelic variations identified by the machine-learning bioinformatics algorithm NetMHC 4.0. Position: residue number (starting from 0); Peptide: Amino acid sequence of the potential ligand

CMV Protein pUL18			
**Position**	**Peptide**	**1-log50k(aff)**	**Affinity (nM)**
253	SEPQCNPLL	0.273	2614.63
322	TMTISSVLL	0.264	2873.80
0	MMTMWCLTL	0.193	6212.24

Supplementary Table 1b: High- and moderate-affinity peptide sequences derived from CMV Protein UL18 that may be presented by HLA-E*01:01 allelic variations identified by the machine-learning bioinformatics algorithm NetMHC 4.0. Position: residue number (starting from 0); Peptide: Amino acid sequence of the potential ligand

CMV Phosphorylated matrix protein pp65 (pUL83)			
**Position**	**Peptide**	**1-log50k(aff)**	**Affinity (nM)**
33	VLPHETRLL	0.330	1405.23
179	VYYTSAFVF	0.214	4939.59
273	VLCPKNMII	0.198	5854.48
304	LCPKSIPGL	0.189	6478.31
14	LGPISGHVL	0.163	8530.14
48	RVSQPSLIL	0.151	9747.11
319	LMNGQQIFL	0.148	100046.59
483	WPPWQAGIL	0.143	10664.68
26	FSRGDTPVL	0.142	10790.14

Supplementary Table 1c: High- and moderate-affinity peptide sequences derived from CMV Phosphorylated matrix protein pp65 (UL83) that may be presented by HLA-E*01:01 allelic variations identified by the machine-learning bioinformatics algorithm NetMHC 4.0. Position: residue number (starting from 0); Peptide: Amino acid sequence of the potential ligand

CMV Immediate-early protein 1 (pUL123)			
**pos**	**Peptide**	**1-log50k(aff)**	**Affinity (nM)**
325	VMLAKRPLI	0.303	1887.52
404	VSPPESPVP	0.203	5571.62
249	IMAYAQKIF	0.168	8151.27
319	VLEETSVML	0.165	8361.18
300	TMYGGISLL	0.134	11774.14

Supplementary Table 1d: High- and moderate-affinity peptide sequences derived from CMV immediate-early protein 1 (UL123) that may be presented by HLA-E*01:01 allelic variations identified by the machine-learning bioinformatics algorithm NetMHC 4.0. Position: residue number (starting from 0); Peptide: Amino acid sequence of the potential ligand

Human leukocyte antigen-G			
**Position**	**Peptide**	**1-log50k(aff)**	**Affinity (nM)**
2	VMAPRTLFL	0.564	112.12
67	RMEPRAPWV	0.439	432.80
3	MAPRTLFLL	0.308	1785.34
10	LLLSGALTL	0.194	6125.67

Supplementary Table 1e: High- and moderate-affinity peptide sequences derived from Human leukocyte antigen G that may be presented by HLA-E*01:01 allelic variations identified by the machine-learning bioinformatics algorithm NetMHC 4.0. Position: residue number (starting from 0); Peptide: Amino acid sequence of the potential ligand

Human leukocyte antigen-Cw*3			
**Position**	**Peptide**	**1-log50k(aff)**	**Affinity (nM)**
2	VMAPRTLIL	0.589	85.69
3	MAPRTLILL	0.217	4799.04
67	RGEPRAPWV	0.187	6597.71
10	LLLSGALAL	0.164	8448.21

Supplementary Table 1f: High- and moderate-affinity peptide sequences derived from Human leukocyte antigen Cw*3 that may be presented by HLA-E*01:01 allelic variations identified by the machine-learning bioinformatics algorithm NetMHC 4.0. Position: residue number (starting from 0); Peptide: Amino acid sequence of the potential ligand

Human heat shock protein 60 (hsp60)			
**Position**	**Peptide**	**1-log50k(aff)**	**Affinity (nM)**
9	QMRPVSRVL	0.276	2530.17
137	VEIRRGVML	0.173	7690.49
53	TMGPKGRTV	0.160	8844.42
32	GADARALML	0.156	9285.31
442	ALLRCIPAL	0.153	9523.60
349	VTKDDAMLL	0.153	9542.06

Supplementary Table 1g: High- and moderate-affinity peptide sequences derived from Human heat shock protein 60 (hsp60) that may be presented by HLA-E*01:01 allelic variations identified by the machine-learning bioinformatics algorithm NetMHC 4.0. Position: residue number (starting from 0); Peptide: Amino acid sequence of the potential ligand

Human immunodeficiency virus 1 Gag protein			
**Position**	**Peptide**	**1-log50k(aff)**	**Affinity (nM)**
256	RMYSPVSIL	0.173	7728.11

Table 4h: High-affinity peptide sequences derived from Human immunodeficiency virus 1 Gag protein that may be presented by HLA-E*01:01 allelic variations identified by the machine-learning bioinformatics algorithm NetMHC 4.0. Position: residue number (starting from 0); Peptide: Amino acid sequence of the potential ligand

Mycobacterium tuberculosis Enoyl-[acyl-carrier-protein] reductase [NADH]			
**Position**	**Peptide**	**1-log50k(aff)**	**Affinity (nM)**
52	RLPAKAPLL	0.547	134.77

Supplementary Table 1i: High-affinity peptide sequences derived from Mycobacterium tuberculosis Enoyl-[acyl-carrier-protein] reductase [NADH] that may be presented by HLA-E*01:01 allelic variations identified by the machine-learning bioinformatics algorithm NetMHC 4.0. Position: residue number (starting from 0); Peptide: Amino acid sequence of the potential ligand
